# Structure cristalline du composé Hg_3-*x*_Sb_*x*_(S+Se)_2+*x*_I_2-*x*_ (*x* ≃ 0.1)

**DOI:** 10.1107/S2056989016002127

**Published:** 2016-02-06

**Authors:** Mohammed Kars, Adrian Gómez Herrero, Thierry Roisnel, Allaoua Rebbah, L. Carlos Otero-Diáz

**Affiliations:** a Université Houari-Boumedienne, Faculté de Chimie, Laboratoire Sciences des Matériaux, BP 32 El-Alia 16111 Bab-Ezzouar, Algeria; b Centro de Microscopia Electrónica, Universidad Complutense, 28040 Madrid, Spain; c Centre de Diffractométrie X, Sciences Chimiques de Rennes, UMR 6226 CNRS Université de Rennes 1, Campus de Beaulieu, Avenue du Général Leclerc, France; d Departomento Inorgánica, Facultad C.C. Químicas, Universidad Complutense, 28040 Madrid, Spain

**Keywords:** crystal structure, mercury chalcohalide, coupled substitution, crankshaft-type

## Abstract

Single crystals of the mercury chalcohalide Hg_3-*x*_Sb_*x*_(S+Se)_2+*x*_I_2-*x*_ (*x* ≃ 0.1) (mercury anti­mony sulfide selenide iodide), were grown by a chemical transport reaction. The structure contains three independent *A* (Hg/Sb) atoms; each atom is strongly covalently bonded with two *X* (Se/S) atoms to form approximately linear *X*–*A*–*X* units. The *X*–*A*–*X* units link to form *A*_4_ *X*_4_ rings, which are combined into infinite crankshaft-type bands running along the [100] direction. Four equatorial *E* (I/*X* = Se,S) atoms at relatively long distances complete the distorted octa­hedral coordination of *A* (Hg/Sb). The crystal under investigation was twinned by non-merohedry with a refined twin domain fraction of 0.814 (6):0.186 (6). The structure is isotypic with Hg_3_Se_2_I_2_ [Beck & Hedderich (2000 ▸). *J. Solid State Chem.* **151**, 73–76], but the current determination reveals a coupled substitution, with partial replacement of Hg^+2^ by Sb^+3^, balanced by the equivalent substitution of I^−1^ by S^−2^ and Se^−2^. Bond-valence calculations are consistent with this relative substitution model.

## Abstract 2

The structure of the title compound is characterized by distorted face-sharing octa­hedra which are combined into infinite crankshaft-type bands running along the [100] direction.

## Contexte chimique   

Les chalcogénohalides au mercure naturel ou synthétique de formulation Hg_3_ *X*_2_ *E*_2_ (*X* = S, Se, Te et *E* = F, Cl, Br, I) ont fait l’objet d’intensives investigations pour leur aptitude à former de nombreux polymorphes (Aurivillius, 1965 ▸; Puff *et al.*, 1966 ▸). La présence de chaînes linéaires *X*–Hg–*X* ainsi que des pyramides trigonales Hg_3_ *X* (*X* = S, Se, Te) en forme de parapluie leur confèrent des motifs de différentes dimensionnalité (Borisov *et al.*, 2001 ▸). Une classification de la plupart de ces composés a été reportée par Pervukhina *et al.* (2003 ▸). Le chalcogenide Hg_3_Se_2_I_2_ a été synthétisé au préalable par Puff (1963 ▸), et sa structure établie après par Beck & Hedderich (2000 ▸).

## Commentaire structurelle   

**Figure 1:**
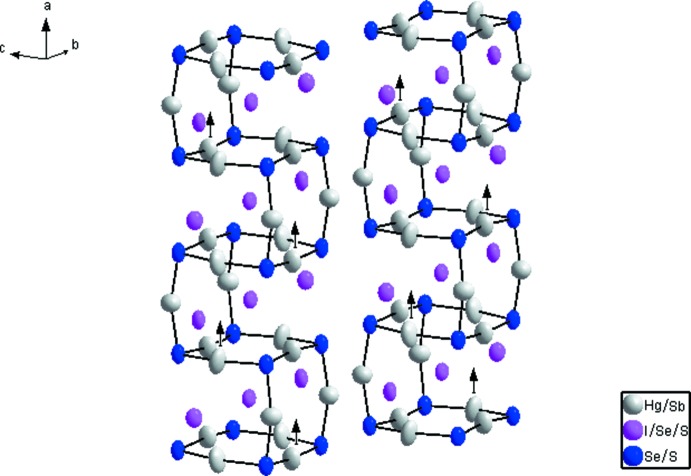
Représentation des liaisons *A—* *X* (*X* = Se, S) agencées en double chaîne infinie en vilebrequin le long de la direction [100], avec un déplacement des ellipsoïdes à 95% de probabilité. Les fléches indiquent le sens dominant de l’agitation thermique.

**Figure 2:**
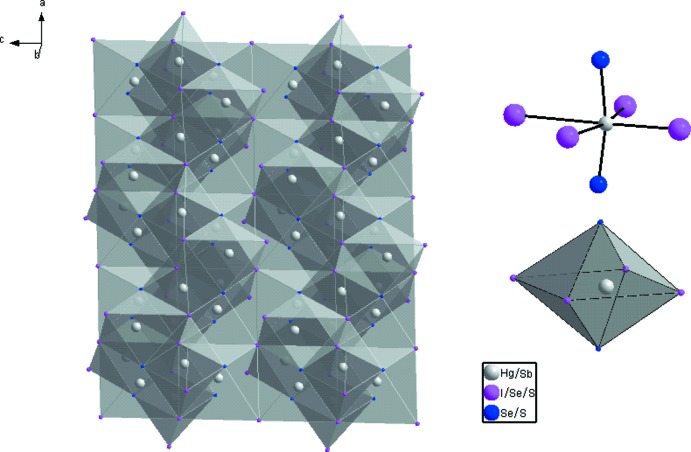
Structure de Hg_3-*x*_Sb_*x*_(S+Se)_2+*x*_I_2-_ *_x_ x* ≃ 0.1 montrant les chaînes en vilebrequin, d’octa­èdres à faces communes des atomes *A* (Hg/Sb) (gauche). Environnement octa­édrique distordu des atomes de *A* (Hg/Sb) (droite).

**Figure 3:**
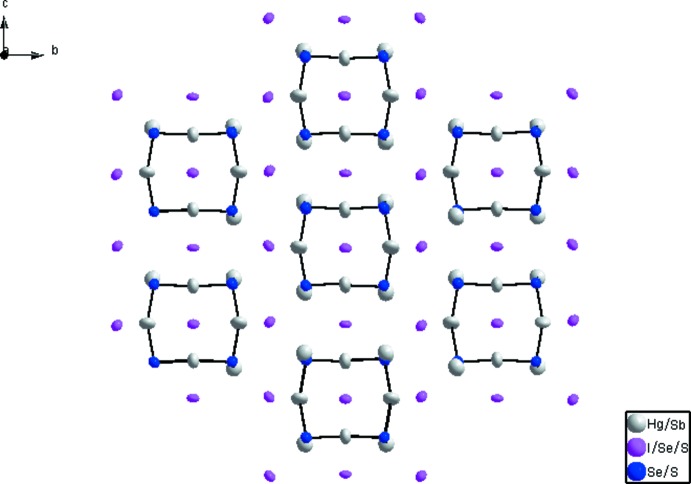
Projection de la structure de Hg_3-*x*_Sb_*x*_(S+Se)_2+*x*_I_2-*x*_ (*x* ≃ 0.1) selon *bc* mettant en évidence la disposition des atomes *E* (I/Se/S), avec un déplacement des ellipsoīdes à 95% de probabilité

Le composé Hg_3-*x*_Sb_*x*_(S+Se)_2+*x*_I_2-_ *_x_ x* ≃ 0.1 appartient à la famille des composés chalcogénides au mercure de formulation Hg_3_ *X*_2_ *E*_2_ (*X* = S, Se et *E* = I, Br, Cl). Comparé aux autres membres, c’est le seul chalcogénide qui présente une substitution couplée, avec un remplacement partiel de Hg^+2^ par Sb^+3^, équilibré par une substitution équivalente de I^−1^ par S^−2^ et Se^−2^. Cette substitution couplée a été déjà observée dans quelques composés minéraux comme la perroudite et la capgaronnite (Mumme & Nickel, 1987 ▸; Mason *et al.*, 1992 ▸). La structure a été décrite au préalable par Beck & Hedderich (2000 ▸), elle est caractérisée par de fortes liaisons covalentes *A*—*X* (*A* = Hg/Sb, *X* = Se/S): 2.4988 (18)-2.5260 (17) Å légèrement inférieure à la somme des rayons covalents: *d*(Hg—Se) = 2.63 Å (Earley, 1950 ▸). Les angles de liaisons *X*—*A*—*X* [160.15 (7)–170.88 (6)°] dévient de la linéarité, ceci est typique des composés chalcogénides au mercure Hg^+2^ (Aurivillius *et al.*, 1965 ▸). Ces liaisons *X*—*A*—*X* agencées en une double chaîne infinie en vilebrequin le long de la direction [100] (Fig. 1 ▸) sont pontées par un autre atome *A*(Hg/Sb)1 engendrant ainsi des anneaux *A*_4_ *X*_4_. La coordination octa­édrique distordue des atomes *A*(Hg/Sb) (Fig. 2 ▸) est complétée par de faibles liaisons avec les atomes *E* = I/*X* = Se,S) placés de part et d’autre de ces chaînes (Fig. 3 ▸). Les distances *A*—*E* (*A* = Hg/Sb, *E* = I/X = Se,S): 3.0377 (19)–3.8824 (19) Å sont proches de la somme des rayons ioniques *d*(Hg_2_^+2^ + I^−1^)= 3.16 Å (Shannon & Prewitt, 1970 ▸), indiquant le caractère ionique de ces liaisons.

Chaque atome *X*(Se/S) se retrouve ainsi lié à trois atomes de *A* pour former des pyramides trigonales *A*_3_ *X* en forme de parapluie avec des angles de liaisons *A*—*X*—*A* entre 96.39 (6)–97.53 (6)° similaires à celles observées dans d’autres composés chalcogénides. Dans cette structure une forte agitation thermique (ADP’s) des atomes *A* à l’exception de *A*(Hg)3 est observée approximativement le long de la direction [100]. En effet *U*_11_(*A*) qui est très prononcée comparé aux autres directions (*U*_22_ et *U*_33_), est environ deux à trois (2–3) fois plus importante que celles observées pour d’autres atomes (*X* et *E*). Cette large ADP’s des atomes *A* qui peut être attribuée simultanément à un effet de désordre statique et dynamique, semble orientée vers l’espace vacant formé par les anneaux *A*_4_ *X*_4_ (fléches Fig. 1 ▸).

## Synthèse et cristallisation   

**Figure 4:**
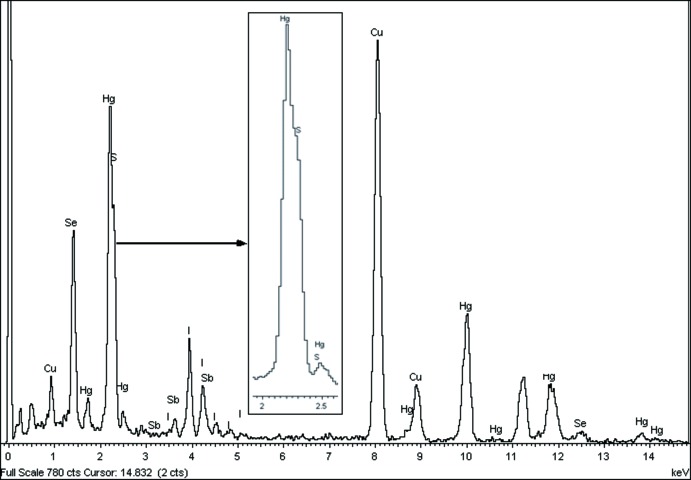
Spectre d’analyse par MET confirmant la présence des cinq éléments chimiques attendus (Hg, I, Sb, S et Se), avec un recouvrement partiel des peaks K-du soufre et M-du mercure.

Les monocristaux de ont été obtenus par transport en phase vapeur à partir d’un mélange approprié des éléments: HgI_2_, I_2_, Sb, S et Se. Le mélange broyé puis scellé dans un tube de quartz est porté à une température de 973 K. Des cristaux de couleur orange sont obtenus après un chauffage d’environ dix jours. L’analyse par MET de plusieurs cristaux qui sont pour la plupart maclés, confirme la présence des 5 éléments chimiques attendus (Hg, I, Sb, S et Se) (Fig. 4 ▸).

## Affinement   

Détails de donnés crystallines, collection de donnés et affinement sont résumés dans le tableau 1 ▸. La structure a été affinée par isotypie à celle de Beck & Hedderich (2000 ▸). Idéalement, et pour un équilibre des charges la substitution de Sb^+3^ des sites Hg^+2^ (à l’exception de Hg3 qui présente un un taux de substitution s.o.f inférieur à 1%) doit s’accompagner par une substitution équivalente de S^−2^ et (ou) Se^−2^ des sites I^−1^. Le meilleur résultat est obtenu pour une distribution partagée des atomes S et Se sur les sites de I, conduisant à la formulation: Hg_3-*x*_Sb_*x*_(S+Se)_2+*x*_I_2-_ *_x_ x* ≃ 0.1. Mais les atomes S et Se sont probablement distribués de manière statistique sur les différents sites de l’iode. De plus, le calcul des valences de liaisons (BVS; Brown 2002 ▸; Brown & Altermatt, 1985 ▸; Brese & O’Keeffe, 1991 ▸) des sites *A*(Hg/Sb) en incluant le taux d’occupation et en utilisant le programme *Valence* (Hormillosa *et al.*, 1993 ▸), conduit à une valeur moyenne des charges de 2.25. Le cristal étudié correspond à une macle non-mériédrique, la fraction en volume des composants est de: 0.814 (6):0.186 (6), et la carte de densité électronique est de ρ_max_ = 1.39 e Å^−3^ [localisée à 1.98 Å de *A*(Hg)3] et ρ_min_ = −2.65 e Å^−3^ [localisée à 1.10 Å de *E*4].

## Supplementary Material

Crystal structure: contains datablock(s) global, I. DOI: 10.1107/S2056989016002127/vn2108sup1.cif

Structure factors: contains datablock(s) I. DOI: 10.1107/S2056989016002127/vn2108Isup2.hkl

CCDC reference: 1451733

Additional supporting information:  crystallographic information; 3D view; checkCIF report

## Figures and Tables

**Table 1:** Détails expérimentaux

Données crystallines	
Formule chimique	Hg_2,899_Sb_0,101_S_0,054_Se_2,038_I_1,908_
*M*_r_	998,6
Système cristallin, groupe d’espace	Orthorhombique, *I* *m* *m* *a*
Température (K)	150
*a*, *b*, *c* (Å)	9,7258 (9), 19,3588 (9), 9,6125 (2)
*V* (Å^3^)	1809,84 (19)
*Z*	8
Type de rayonnement	Mo *K*α
μ (mm^−1^)	63,99
Crystal size (mm)	0,18 × 0,09 × 0,06

Collection de données	
Diffractomètre	Bruker APEXII
Correction d’absorption	Multi-scan (*TWINABS*; Bruker, 2006 ▸)
*T*_min_, *T*_max_	0,006, 0,020
Nombre de réflexions mesurées, indépendantes et observées [*I* > 2σ(*I*)]	7021, 1628, 1031
*R*_int_	0,069
(sin θ/λ)_max_ (Å^−1^)	0,806

Affinement	
*R*[*F* ^2^ > 3σ(*F*)], *wR*(*F*), *S*	0,048, 0,064, 1,44
Nombre de réflexions	1628
Nombre de paramètres	48
Δρ_max_, Δρ_min_ (e Å^−3^)	1,98, −2,65

Programmes informatiques: *APEX2*, *SAINT* et *CELL_NOW* (Bruker, 2006 ▸), *SIR97* (Altomare *et al.*, 1999 ▸), *JANA2006* (Petříček *et al.*, 2014 ▸) et *DIAMOND* (Brandenburg & Putz, 2009 ▸).

## supplementary crystallographic information

### Crystal data

**Table:**

Hg_2.899_Sb_0.101_S_0.054_Se_2.038_I_1.908_	*F*(000) = 3267
*M**_r_* = 998.6	*D*_x_ = 7.327 Mg m^−^^3^
Orthorhombic, *I**m**m**a*	Mo *K*α radiation, λ = 0.71073 Å
Hall symbol: -I 2b 2	Cell parameters from 1893 reflections
*a* = 9.7258 (9) Å	θ = 3.0–33.8°
*b* = 19.3588 (9) Å	µ = 63.99 mm^−^^1^
*c* = 9.6125 (2) Å	*T* = 150 K
*V* = 1809.84 (19) Å^3^	Block, orange
*Z* = 8	0.18 × 0.09 × 0.06 mm

### Data collection

**Table:**

Bruker APEXII diffractometer	1628 independent reflections
Radiation source: X-ray tube	1031 reflections with *I* > 3σ(*I*)
Graphite monochromator	*R*_int_ = 0.069
CCD rotation images, thin slices scans	θ_max_ = 35.0°, θ_min_ = 2.1°
Absorption correction: multi-scan (*TWINABS*; Bruker, 2006)	*h* = −14→15
*T*_min_ = 0.006, *T*_max_ = 0.020	*k* = −31→27
7021 measured reflections	*l* = −15→12

### Refinement

**Table:**

Refinement on *F*	0 restraints
*R*[*F*^2^ > 2σ(*F*^2^)] = 0.048	34 constraints
*wR*(*F*^2^) = 0.064	Weighting scheme based on measured s.u.'s *w* = 1/(σ^2^(*F*) + 0.0001*F*^2^)
*S* = 1.44	(Δ/σ)_max_ = 0.0001
1628 reflections	Δρ_max_ = 1.98 e Å^−^^3^
48 parameters	Δρ_min_ = −2.65 e Å^−^^3^

### Special details

**Table:**

Refinement. The refinement was carried out against all reflections. The conventional **R**-factor is always based on **F**. The goodness of fit as well as the weighted **R**-factor are based on **F** and **F**^2^ for refinement carried out on **F** and **F**^2^, respectively. The threshold expression is used only for calculating **R**-factors *etc*. and it is not relevant to the choice of reflections for refinement. The crystal studied was twinned with a refined twin domain fraction of 0.814 (6):0.186 (6). The overlaps of reflection between the twin domains were calculated by Jana2006 software using the twinning matrix and user- defined treshold 0.1° for full overlap and 0.3° for full separation. Due to no support for twinning in the official CIF dictionary the twinning matrix has been saved in the CIF file using special _jana_cell_twin_matrix keyword. The program used for refinement, *Jana2006*, uses the weighting scheme based on the experimental expectations, see _refine_ls_weighting_details, that does not force **S** to be one. Therefore the values of **S** are usually larger than the ones from the *SHELX* program.

### Fractional atomic coordinates and isotropic or equivalent isotropic displacement parameters (Å^2^)

**Table:**

	*x*	*y*	*z*	*U*_iso_*/*U*_eq_	Occ. (<1)
Hg1	0.22648 (9)	0.25	−0.00243 (7)	0.0283 (3)	0.935 (14)
Sb1	0.22648 (9)	0.25	−0.00243 (7)	0.0283 (3)	0.065 (14)
Hg2	0.25	0.09884 (5)	−0.25	0.0347 (3)	0.964 (13)
Sb2	0.25	0.09884 (5)	−0.25	0.0347 (3)	0.036 (13)
Hg3	0.5	0.11573 (5)	0.04687 (10)	0.0312 (2)	
Se1	0.24364 (14)	0.12133 (9)	0.00877 (18)	0.0211 (6)	0.98 (2)
S1	0.24364 (14)	0.12133 (9)	0.00877 (18)	0.0211 (6)	0.02 (2)
I1	0	0.25	0.7532 (2)	0.0181 (7)	0.91 (3)
Se2	0	0.25	0.7532 (2)	0.0181 (7)	0.09 (3)
I2	0	0.25	0.2488 (2)	0.0187 (7)	0.94 (3)
Se3	0	0.25	0.2488 (2)	0.0187 (7)	0.06 (3)
I3	0	0.00089 (5)	0.2378 (2)	0.0207 (6)	0.983 (12)
S2	0	0.00089 (5)	0.2378 (2)	0.0207 (6)	0.017 (12)

### Atomic displacement parameters (Å^2^)

**Table:**

	*U*^11^	*U*^22^	*U*^33^	*U*^12^	*U*^13^	*U*^23^
Hg1	0.0400 (5)	0.0168 (4)	0.0282 (5)	0	0.0001 (3)	0
Sb1	0.0400 (5)	0.0168 (4)	0.0282 (5)	0	0.0001 (3)	0
Hg2	0.0522 (5)	0.0349 (5)	0.0169 (4)	0	−0.0065 (3)	0
Sb2	0.0522 (5)	0.0349 (5)	0.0169 (4)	0	−0.0065 (3)	0
Hg3	0.0271 (3)	0.0329 (4)	0.0335 (5)	0	0	0.0027 (3)
Se1	0.0304 (10)	0.0166 (11)	0.0164 (11)	−0.0007 (8)	0.0001 (4)	0.0013 (4)
S1	0.0304 (10)	0.0166 (11)	0.0164 (11)	−0.0007 (8)	0.0001 (4)	0.0013 (4)
I1	0.0230 (9)	0.0195 (15)	0.0118 (11)	0	0	0
Se2	0.0230 (9)	0.0195 (15)	0.0118 (11)	0	0	0
I2	0.0275 (9)	0.0203 (15)	0.0082 (10)	0	0	0
Se3	0.0275 (9)	0.0203 (15)	0.0082 (10)	0	0	0
I3	0.0284 (8)	0.0160 (13)	0.0175 (11)	0	0	−0.0039 (3)
S2	0.0284 (8)	0.0160 (13)	0.0175 (11)	0	0	−0.0039 (3)

### Bond lengths (Å)

**Table:**

Hg1—Sb1	0	Hg2—S1^vi^	2.5260 (17)
Hg1—Hg2	3.7788 (8)	Hg2—I1^iv^	3.8048 (8)
Hg1—Hg2^i^	3.7788 (8)	Hg2—I1^v^	3.8048 (8)
Hg1—Sb2	3.7788 (8)	Hg2—Se2^iv^	3.8048 (8)
Hg1—Sb2^i^	3.7788 (8)	Hg2—Se2^v^	3.8048 (8)
Hg1—Hg3	3.7493 (10)	Hg2—I3^vii^	3.1069 (9)
Hg1—Hg3^ii^	3.7493 (10)	Hg2—I3^viii^	3.1069 (9)
Hg1—Se1	2.4988 (18)	Hg2—S2^vii^	3.1069 (9)
Hg1—Se1^iii^	2.4988 (18)	Hg2—S2^viii^	3.1069 (9)
Hg1—S1	2.4988 (18)	Sb2—Hg3	3.7633 (8)
Hg1—S1^iii^	2.4988 (18)	Sb2—Hg3^vi^	3.7633 (8)
Hg1—I1^iv^	3.2206 (19)	Sb2—Se1	2.5260 (17)
Hg1—I1^v^	3.5896 (18)	Sb2—Se1^vi^	2.5260 (17)
Hg1—Se2^iv^	3.2206 (19)	Sb2—S1	2.5260 (17)
Hg1—Se2^v^	3.5896 (18)	Sb2—S1^vi^	2.5260 (17)
Hg1—I2	3.2686 (18)	Sb2—I1^iv^	3.8048 (8)
Hg1—I2^v^	3.6084 (17)	Sb2—I1^v^	3.8048 (8)
Hg1—Se3	3.2686 (18)	Sb2—Se2^iv^	3.8048 (8)
Hg1—Se3^v^	3.6084 (17)	Sb2—Se2^v^	3.8048 (8)
Sb1—Hg2	3.7788 (8)	Sb2—I3^vii^	3.1069 (9)
Sb1—Hg2^i^	3.7788 (8)	Sb2—I3^viii^	3.1069 (9)
Sb1—Sb2	3.7788 (8)	Sb2—S2^vii^	3.1069 (9)
Sb1—Sb2^i^	3.7788 (8)	Sb2—S2^viii^	3.1069 (9)
Sb1—Hg3	3.7493 (10)	Hg3—Se1	2.5224 (14)
Sb1—Hg3^ii^	3.7493 (10)	Hg3—Se1^ix^	2.5224 (14)
Sb1—Se1	2.4988 (18)	Hg3—S1	2.5224 (14)
Sb1—Se1^iii^	2.4988 (18)	Hg3—S1^ix^	2.5224 (14)
Sb1—S1	2.4988 (18)	Hg3—I1^v^	3.8824 (19)
Sb1—S1^iii^	2.4988 (18)	Hg3—Se2^v^	3.8824 (19)
Sb1—I1^iv^	3.2206 (19)	Hg3—I2^v^	3.2579 (16)
Sb1—I1^v^	3.5896 (18)	Hg3—Se3^v^	3.2579 (16)
Sb1—Se2^iv^	3.2206 (19)	Hg3—I3^vii^	3.731 (2)
Sb1—Se2^v^	3.5896 (18)	Hg3—I3^v^	3.0377 (19)
Sb1—I2	3.2686 (18)	Hg3—S2^vii^	3.731 (2)
Sb1—I2^v^	3.6084 (17)	Hg3—S2^v^	3.0377 (19)
Sb1—Se3	3.2686 (18)	Se1—S1	0
Sb1—Se3^v^	3.6084 (17)	Se1—I3	3.987 (2)
Hg2—Sb2	0	Se1—S2	3.987 (2)
Hg2—Hg3	3.7633 (8)	S1—I3	3.987 (2)
Hg2—Hg3^vi^	3.7633 (8)	S1—S2	3.987 (2)
Hg2—Se1	2.5260 (17)	I1—Se2	0
Hg2—Se1^vi^	2.5260 (17)	I2—Se3	0
Hg2—S1	2.5260 (17)	I3—S2	0

Symmetry codes: (i) −*x*+1/2, −*y*+1/2, −*z*−1/2; (ii) −*x*+1, −*y*+1/2, *z*; (iii) *x*, −*y*+1/2, *z*; (iv) *x*, *y*, *z*−1; (v) −*x*+1/2, *y*, −*z*+1/2; (vi) −*x*+1/2, *y*, −*z*−1/2; (vii) −*x*+1/2, −*y*, *z*−1/2; (viii) *x*, −*y*, −*z*; (ix) −*x*+1, *y*, *z*.
